# Sub-classification of Stage II colorectal cancer based on clinicopathological risk factors for recurrence

**DOI:** 10.1007/s00595-013-0807-y

**Published:** 2013-12-21

**Authors:** Takuzo Hashimoto, Michio Itabashi, Shinpei Ogawa, Tomoichiro Hirosawa, Yoshiko Bamba, Satoru Shimizu, Shingo Kameoka

**Affiliations:** 1 Department of Surgery II, Tokyo Women’s Medical University, 8-1 Kawadacho, Shinjuku-ku, Tokyo, 162-8666 Japan; 2 Tokyo Women’s Medical University Medical Research Institute, Tokyo, Japan

**Keywords:** Stage II colorectal cancer, Clinicopathological factors, Risk factors for recurrence, Sub-classification

## Abstract

**Purpose:**

To make a Stage II colorectal cancer (CRC) sub-classification based on clinicopathological factors.

**Methods:**

The subjects of this study were 422 patients with Stage II CRC, who underwent curative surgery with dissection of more than 12 lymph nodes. We used the logistic regression analysis or model and Cox’s proportional hazard regression model for analysis.

**Results:**

Preoperative carcinoembryonic antigen (CEA) level (*p* = 0.0057), macroscopic type (*p* = 0.0316), and depth of invasion (*p* = 0.0401) were extracted as independent risk factors for recurrence, whereas the preoperative CEA level (*p* = 0.0045) and depth of invasion (*p* = 0.0395) were extracted as independent predictors of 5-year disease-free survival. We defined depth of invasion (pT4) and the preoperative CEA level (abnormal) as risk factors for recurrence, and classified Grade A as a normal CEA level regardless of depth invasion, Grade B as depth of invasion to pT3 and an elevated CEA level, and Grade C as depth of invasion to pT4 and an elevated CEA level. There were significant differences in cumulative 5-year disease-free survival rates among each grade (Grade A vs. Grade B, *p* = 0.0474; Grade A vs. Grade C, *p* < 0.0001; Grade B vs. Grade C, *p* = 0.0134).

**Conclusion:**

The sub-classification of Stage II CRC, according not only to depth of invasion but also to preoperative CEA level, is important for predicting the prognosis.

## Introduction

The morbidity associated with colorectal cancer (CRC) is increasing in Japan. Despite advances in chemotherapy and surgical techniques, the recurrence rate increases as the stage of the cancer advances. According to the national registry for CRC, the 5-year cumulative survival rates after curative surgery are 94.3 % for Stage 0, 90.6 % for Stage I, 81.2 % for Stage II, 71.4 % for Stage IIIa, and 56.0 % for Stage IIIb [1].

For Stage II and Stage III CRC, postoperative adjuvant chemotherapy is integral for managing metastatic recurrence, whereas for Stage 0 and Stage I CRC, successful curative surgery is likely to be achieved. It has been established that for Stage III CRC, surgery with adjuvant chemotherapy results in a better prognosis than surgery alone [2–4]. However, no consensus has been reached on the effectiveness of adjuvant chemotherapy for Stage II CRC [5]. European and American guidelines suggest selecting those patients at high risk of recurrence and, taking into consideration the risks and benefits, once informed consent for adjuvant chemotherapy after the surgery is obtained, recommend the same treatment and duration as for Stage III CRC [6, 7]. The Japanese guidelines also state that since the efficacy of adjuvant chemotherapy for Stage II has not yet been established, it is not suited for all cases and its use must be selective [8]. However, there are no specific reports on high-risk recurrence factors. We tried to establish a variant of the Stage II subgroup by selecting clinicopathological factors related to the risk of recurrence, and investigated the efficacy of adaptations to adjuvant chemotherapy for Stage II CRC.

## Patients and methods

The subjects of this study were 422 patients with Stage II CRC treated by radical surgery, with more than 12 lymph nodes dissected, in our Department of Surgery between 1987 and 2008, excluding those with hetero/chronic cancers and colitic cancer. According to the Japanese classification of Colorectal Carcinoma; Second English Edition, the tumor was localized in the colon, including the rectosigmoid colon, in 344 patients; and in the rectum in 78 patients [9]. First, we calculated the recurrence risk from clinicopathological factors according to recurrence rates and cumulative 5-year disease-free survival rates (5y-DFS), and created a sub-classification of Stage II based on a combination of the risk factors. The χ^2^ test and Log-rank test were used for statistical univariate analysis, and a logistic regression analysis or model and Cox’s proportional hazard regression model were used for statistical multivariate analysis. A *p* value of less than 0.05 was taken to indicate significance (JMP^®^ ver. 8.0.1 statistics system).

## Results

### Evaluating risk factors according to recurrence rate and 5y-DFS

Recurrence rate, age, preoperative carcinoembryonic antigen (CEA) level, macroscopic type, and depth of invasion were extracted by univariate analysis, while preoperative CEA levels, macroscopic type and depth of invasion were the independent factors extracted by multivariate analysis (Table 1). Furthermore, the 5y-DFS, preoperative CEA levels, macroscopic type, and depth of invasion were extracted by univariate analysis, while preoperative CEA levels and depth of invasion were independent factors extracted by multivariate analysis (Table 2). We defined the extracted common independent factors; namely, preoperative CEA levels and depth of invasion, as Stage II risk factors of recurrence.

**Table 1:** Statistical analysis of clinicopathological risk factors according to recurrence rates

		Recurrence	Total	*p* (univariate)	*p* (multivariate)
Gender	(Male/female)	41/21	252/170	NS	
Age	(<60 years/≥60 years)	29/33	147/275	0.0427	NS
CEA	(Normal/abnormal)	30/25	274/106	0.0031	0.0057
Locus	(Colon/rectum)	50/12	344/78	NS	
Macroscopic type	(Type 0,1,2/type 3,4,5)	48/14	363/54	0.0225	0.0316
Size	(<70 mm/≥70 mm)	43/18	302/111	NS	
Circumference	(<1/3//2/≥3)	6/54	37/361	NS	
Histopathological type	(tub1, tub2/por)	60/2	391/30	NS	
Depth of invasion	(~pT3/pT4~)	42/20	341/81	0.0081	0.0401
Lymphatic invasion	(~ly1/ly2~)	48/13	354/67	NS	
Venous invasion	(~v1/v2~)	58/3	405/15	NS	
Ileus and perforation	(Presence/absence)	4/57	23/386	NS

**Table 2:** Statistical analysis of clinicopathological risk factors according to cumulative 5-year disease-free survival rates

		5 years-DFS (%)	*p* (Logrank)	Exp	*p* (Cox hazard)
Gender	(Male/female)	81.7/86.7	NS		
Age	(<60 years/≥60 years)	90.0/85.3	NS		
CEA	(Normal/abnormal)	88.3/72.4	0.0010	2.22	0.0045
Locus	(Colon/rectum)	84.0/82.7	NS		
Macroscopic type	(Type 0,1,2/type 3,4,5)	84.5/73.9	0.0199		NS
Size	(<70 mm/≥70 mm)	84.4/81.7	NS		
Circumference	(<1/3//2/≥3)	84.0/83.3	NS		
Histopathological type	(tub1, tub2/por)	83.1/91.6	NS		
Depth of invasion	(pT3/pT4)	86.2/72.1	0.0081	0.53	0.0395
Lymphatic invasion	(ly0,1/ly2,3)	84.8/77.5	NS		
Venous invasion	(v0,1/v2,3)	84.2/79.4	NS		
Ileus and perforation	(Presence/absence)	84.2/83.5	NS		

### Trial for Stage II sub-classification

**Fig. 1:**
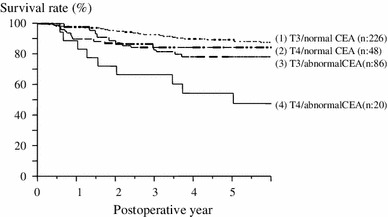
5-year disease-free survival curve according to the combination of depth of invasion and preoperative CEA levels. There was no significant difference between either T3 or T4 depth of invasion and normal CEA levels (*p* = 0.4498)

**Fig. 2:**
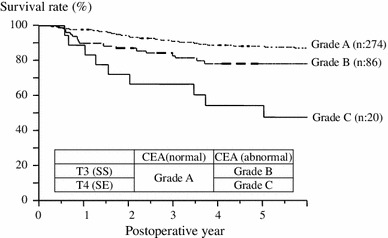
The sub-classification 5-year disease-free survival curve according to the combination of depth of invasion and the preoperative CEA level. Grade A, a normal CEA level, regardless of depth invasion; Grade B, pT3 depth of invasion and an abnormal CEA level; Grade C, pT4 depth of invasion and an abnormal CEA level. There was a significant difference among each grade (Grade A vs. Grade B: *p* = 0.0474, Grade A vs. Grade C; *p* < 0.0001, Grade B vs. Grade C; *p* = 0.0134)

We investigated the 5y-DFS of Stage II based on a combination of the risk factors of recurrence. There was no significant difference between depth of invasion (T3) and a normal CEA or between depth of invasion (T4) and a normal CEA (*p* = 0.4498; Fig. 1). We classified Grade A as a normal CEA level, regardless of depth of invasion, Grade B as depth of invasion (pT3) and an abnormal preoperative CEA level, and Grade C as depth of invasion (pT4) and an abnormal preoperative CEA level. There was a significant difference in the cumulative 5-year disease-free survival rate among each grade (Grade A vs. Grade B: *p* = 0.0474, Grade A vs Grade C; *p* < 0.0001, Grade B vs. Grade C; *p* = 0.0134; Fig. 2).

## Discussion

**Fig. 3:**
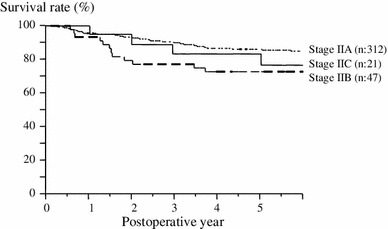
The 5-year disease-free survival curve according to the TNM classification 7th edition. There was a significant difference between stage IIA and stage IIB CRC (*p* = 0.0252), but no significant differences between stage IIB and IIC (*p* = 0.5857) or Stage IIA and IIC CRC (*p* = 0.4314)

It is important to establish the optimum form of postoperative follow-up and find the best adjuvant chemo therapy for patients who undergo surgery for Stage II or Stage III CRC, because of the risk of metastasis or recurrence. The management of these patients differs from that of those with Stage 0 or Stage I CRC, which is curable. Generally, adjuvant chemotherapy for 6–8 months is recommended for Stage III CRC [10], but it should not be administered routinely for Stage II CRC and carefully considered according to selected risk factors for recurrence [11–13]. CRC is classified as Stage II or Stage III by the presence of lymph node metastasis. We tried to exclude the possibility of stage migration between Stage II and Stage III and in this study we investigated 422 Stage II CRC cases with more than 12 lymph nodes dissected. While this study focused on cases of more than 12 lymph nodes, there were no significant differences in recurrence (*p* = 0.8130) and overall survival (*p* = 0.4499) rates between the “more than 12 lymph node dissection” group and the “less than 12 lymph node dissection” group. However, as the data show a poor prognosis when less than 12 lymph nodes were dissected, we think that a lymph node count of less than 12 is one of the recurrence risk factors of Stage II CRC. Preoperative CEA levels and depth of invasion were extracted as significant independent factors for recurrence, and the 5y-DFS. Stage II CRC has no lymph node metastasis and is classified only by depth of invasion. The TNM classification 6th edition classifies depth of invasion into IIA (depth invasion T3) and IIB (depth invasion T4), and the 7th edition classifies it into Stage IIA (depth of invasion T3), IIB (depth of invasion T4a), and IIC (depth of invasion T4b) [14, 15]. We considered preoperative CEA levels as a sub-classification factor, similarly to depth of invasion. We defined Grade A as normal preoperative CEA levels, regardless of depth of invasion, Grade B as abnormal preoperative CEA levels and depth of invasion T3, and Grade C as abnormal preoperative CEA levels and depth of invasion T4. Thus, it may be possible to distinguish the risk of recurrence. We tried to apply the findings of this study to the TNM classification 7th edition, examining 5y-DFS, and found a significant difference between Stage IIA and Stage IIB CRC (*p* = 0.0252), but no significant differences between Stage IIB and IIC CRC (*p* = 0.5857) or Stage IIA and IIC CRC (*p* = 0.4314; Fig. 3). In an attempt to explain these results, Stage IIA comprised 226 Grade A cases and 86 Grade B cases affected by CEA factors besides depth of invasion. It has been proposed that adjuvant chemotherapy results in 3–5 % extra improvement in the survival rate of patients with Stage II CRC [16, 17]. Selecting the Stage II CRC group at highest risk of recurrence should be simple and this sub-classification is useful for preoperative evaluation of the risk of recurrence. However, prospective studies are needed for resolving this sub-classification.

In conclusion, by reviewing the clinicopathological factors, this study found preoperative CEA levels (abnormal) and depth of invasion (pT4) to be risk factors of recurrence in patients with Stage II CRC. This sub-classification of Stage II CRC according to the T factor and the preoperative CEA level is useful for predicting prognosis. More attention should be paid to recurrence risk in patients with a high preoperative CEA level even if the depth of invasion is T3.
